# Supplementary material for: Temporal distribution shifts of Chum salmon (Oncorhynchus keta) with sea surface temperature changes at their southern limit in the North Pacific
Source: PLoS One. 2025 Feb 26;20(2):e0317917. doi: 10.1371/journal.pone.0317917 (PMC11864555; doi:10.1371/journal.pone.0317917)
Supplement: S1 Table — (DOCX) [file pone.0317917.s001.docx]

| Type 1 | Type 2 | Type 3 |
| --- | --- | --- |
| 2006 | 2009 | 2007 |
| 2008 | 2011 | 2010 |
| 2018 | 2013 | 2012 |
|  | 2015 | 2014 |
|  | 2016 | 2017 |
