# Supplementary material for: Temporal distribution shifts of Chum salmon (Oncorhynchus keta) with sea surface temperature changes at their southern limit in the North Pacific
Source: PLoS One. 2025 Feb 26;20(2):e0317917. doi: 10.1371/journal.pone.0317917 (PMC11864555; doi:10.1371/journal.pone.0317917)
Supplement: S2 Table — (DOCX) [file pone.0317917.s002.docx]

| **Type** | **Region** | **AIC_poly** | **AIC_exp** | **AIC_logistic** |
| --- | --- | --- | --- | --- |
| **T1** | CR1 | −1690.54 | −1675.80 | −1671.52 |
|  | CR2 | −1583.90 | −1573.74 | −1573.75 |
|  | CR3 | −1382.67 | −1371.49 | −1371.50 |
|  | CR4 | −1570.43 | −1543.16 | −1543.08 |
|  | CR5 | −645.57 | −644.34 | −644.30 |
|  | CR6 | −105.52 | −107.38 | −107.38 |
| **T2** | CR1 | −3279.02 | −3203.97 | −3205.73 |
|  | CR2 | −3160.32 | −3125.87 | −3126.45 |
|  | CR3 | −2956.34 | −2894.24 | −2894.25 |
|  | CR4 | −2416.48 | −2387.91 | −2387.08 |
|  | CR5 | −1526.46 | −1509.65 | −1509.70 |
|  | CR6 | −787.77 | −781.63 | −781.66 |
| **T3** | CR1 | −3939.66 | −3830.90 | −3833.00 |
|  | CR2 | −3371.27 | −3355.03 | −3355.27 |
|  | CR3 | −2601.19 | −2569.77 | −2570.45 |
|  | CR4 | −2191.97 | −2176.11 | −2176.39 |
|  | CR5 | −1286.14 | −1278.56 | −1278.62 |
|  | CR6 | −548.86 | −542.96 | −543.01 |
