# Supplementary material for: Temporal distribution shifts of Chum salmon (Oncorhynchus keta) with sea surface temperature changes at their southern limit in the North Pacific
Source: PLoS One. 2025 Feb 26;20(2):e0317917. doi: 10.1371/journal.pone.0317917 (PMC11864555; doi:10.1371/journal.pone.0317917)
Supplement: S3 Table — (DOCX) [file pone.0317917.s003.docx]

| **Type** | **Region** | **AIC_poly** | **AIC_exp** | **AIC_logistic** |
| --- | --- | --- | --- | --- |
| **T1** | CR1 | 864.05 | 863.24 | 863.32 |
|  | CR2 | 1042.31 | 1043.63 | 1043.66 |
|  | CR5 | 285.21 | 282.94 | 282.23 |
|  | CR6 | 442.10 | 413.68 | 413.64 |
| **T2** | CR1 | 708.51 | 710.40 | 710.23 |
|  | CR2 | 2011.68 | 1951.29 | 1951.97 |
|  | CR5 | 669.34 | 658.79 | 658.82 |
|  | CR6 | 802.65 | 792.49 | 792.53 |
| **T3** | CR1 | 1350.16 | 1346.48 | 1346.58 |
|  | CR2 | 1865.71 | 1855.95 | 1853.98 |
|  | CR5 | 268.02 | 265.52 | 264.22 |
|  | CR6 | 639.46 | 622.25 | 622.35 |
