# Supplementary material for: Temporal distribution shifts of Chum salmon (Oncorhynchus keta) with sea surface temperature changes at their southern limit in the North Pacific
Source: PLoS One. 2025 Feb 26;20(2):e0317917. doi: 10.1371/journal.pone.0317917 (PMC11864555; doi:10.1371/journal.pone.0317917)
Supplement: S4 Table — (DOCX) [file pone.0317917.s004.docx]

| **Type** | **Region** | ***b_1_*** | ***b_2_*** | ***b_3_*** | **MSE** |
| --- | --- | --- | --- | --- | --- |
| **T1** | CR1 | −0.230^***^ | 0.027^***^ | −0.001^***^ | 0.001 |
|  | CR2 | −0.234^**^ | 0.028^***^ | −0.001^***^ | 0.001 |
|  | CR3 | −0.345^***^ | 0.037^***^ | −0.001^***^ | 0.002 |
|  | CR4 | −0.555^***^ | 0.058^***^ | −0.001^***^ | 0.001 |
|  | CR5 | −1.977^*^ | 0.200^*^ | −0.005^*^ | 0.004 |
|  | CR6 | 1.449 | −0.108 | 0.002 | 0.006 |
| **T2** | CR1 | −0.198^***^ | 0.024^***^ | −0.001^***^ | 0.000 |
|  | CR2 | −0.281^***^ | 0.033^***^ | −0.001^***^ | 0.001 |
|  | CR3 | −0.428^***^ | 0.047^***^ | −0.001^***^ | 0.001 |
|  | CR4 | −0.506^***^ | 0.051^***^ | −0.001^***^ | 0.001 |
|  | CR5 | −1.396^***^ | 0.134^***^ | −0.003^***^ | 0.002 |
|  | CR6 | −0.965^**^ | 0.097^**^ | −0.002^**^ | 0.005 |
| **T3** | CR1 | −0.188^***^ | 0.023^***^ | −0.001^***^ | 0.000 |
|  | CR2 | −0.114^***^ | 0.014^***^ | 0.000^***^ | 0.001 |
|  | CR3 | −0.256^***^ | 0.030^***^ | −0.001^***^ | 0.001 |
|  | CR4 | −0.391^***^ | 0.042^***^ | −0.001^***^ | 0.001 |
|  | CR5 | −1.072^**^ | 0.110^**^ | −0.003^**^ | 0.003 |
|  | CR6 | −1.245^**^ | 0.126^**^ | −0.003^**^ | 0.002 |

* : p < 0.1, ** : p < 0.05, *** : p < 0.001
