# Supplementary material for: Temporal distribution shifts of Chum salmon (Oncorhynchus keta) with sea surface temperature changes at their southern limit in the North Pacific
Source: PLoS One. 2025 Feb 26;20(2):e0317917. doi: 10.1371/journal.pone.0317917 (PMC11864555; doi:10.1371/journal.pone.0317917)
Supplement: S5 Table — (DOCX) [file pone.0317917.s005.docx]

| **Type** | **Region** | ***b_1_*** | ***b_2_*** | ***b_3_*** | **MSE** |
| --- | --- | --- | --- | --- | --- |
| **T1** | CR1 | −0.216 | 0.032 | −0.001 | 0.002 |
|  | CR2 | −0.035 | 0.009 | 0.000 | 0.001 |
|  | CR5 | −2.436^*^ | 0.257^*^ | −0.007^*^ | 0.000 |
|  | CR6 | −1.382^***^ | 0.151^***^ | −0.004^***^ | 0.000 |
| **T2** | CR1 | 0.131 | −0.015 | 0.001 | 0.006 |
|  | CR2 | −0.565^***^ | 0.071^***^ | −0.002^***^ | 0.000 |
|  | CR5 | −2.193^***^ | 0.227^***^ | −0.006^***^ | 0.000 |
|  | CR6 | −2.091^***^ | 0.227^***^ | −0.006^***^ | 0.001 |
| **T3** | CR1 | −0.457^**^ | 0.060^**^ | −0.002^**^ | 0.003 |
|  | CR2 | −0.271^***^ | 0.034^***^ | −0.001^***^ | 0.001 |
|  | CR5 | −1.152^*^ | 0.124^*^ | −0.003^*^ | 0.000 |
|  | CR6 | −1.364^***^ | 0.147^***^ | −0.004^***^ | 0.000 |

* : p < 0.1, ** : p < 0.05, *** : p < 0.001
