# Supplementary figures and images for: Temporal distribution shifts of Chum salmon (Oncorhynchus keta) with sea surface temperature changes at their southern limit in the North Pacific
Source: PLoS One. 2025 Feb 26;20(2):e0317917. doi: 10.1371/journal.pone.0317917 (PMC11864555; doi:10.1371/journal.pone.0317917)

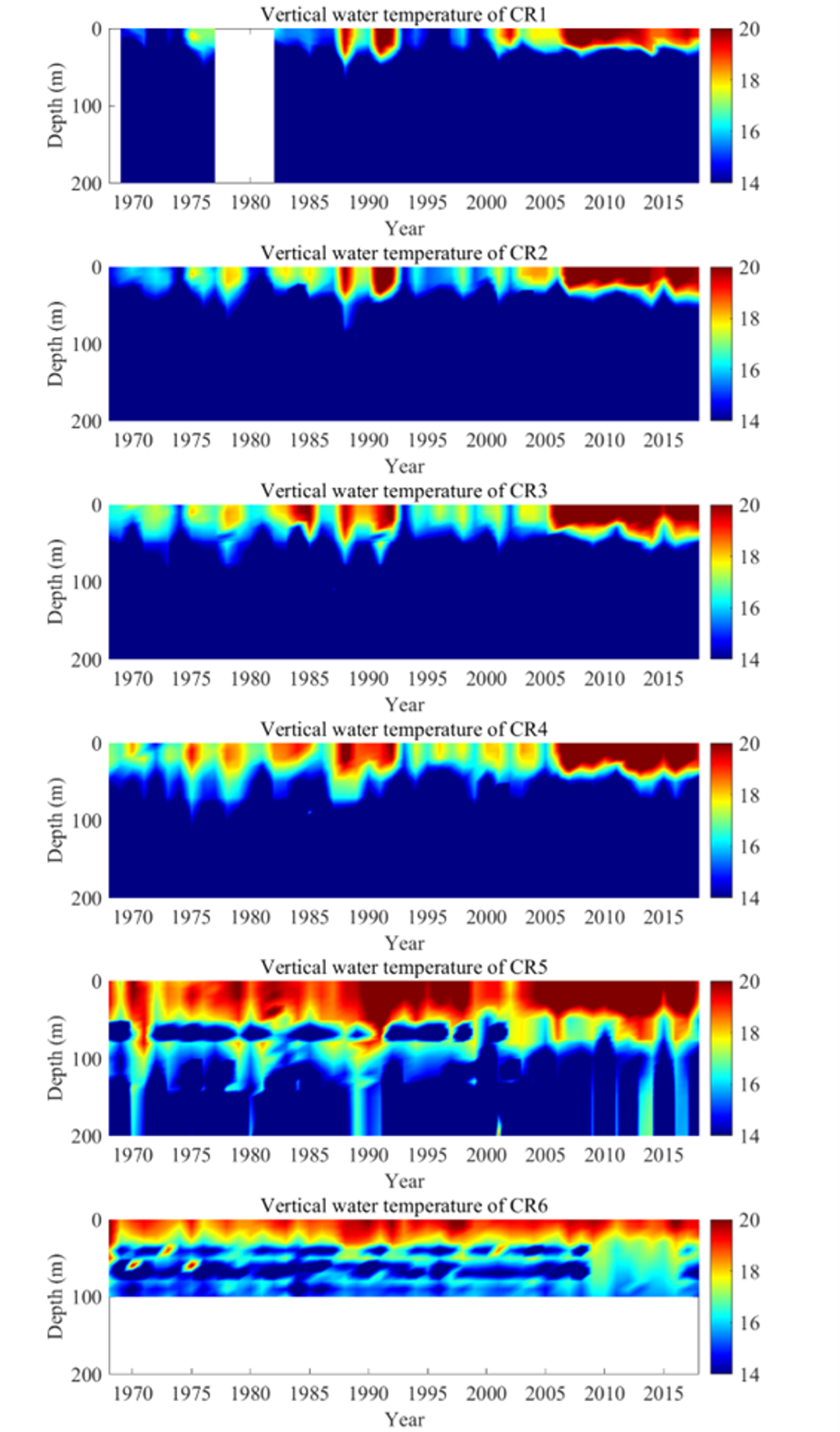

Supplement: S1 Fig — The x-axis represents the years, the y-axis represents the depth, and the colors indicate temperature. The temperature range is set to 14–20 °C, which corresponds to the optimal temperature range for Chum migration during the spawning season in the ESCK. (TIF) [file pone.0317917.s006.TIF]

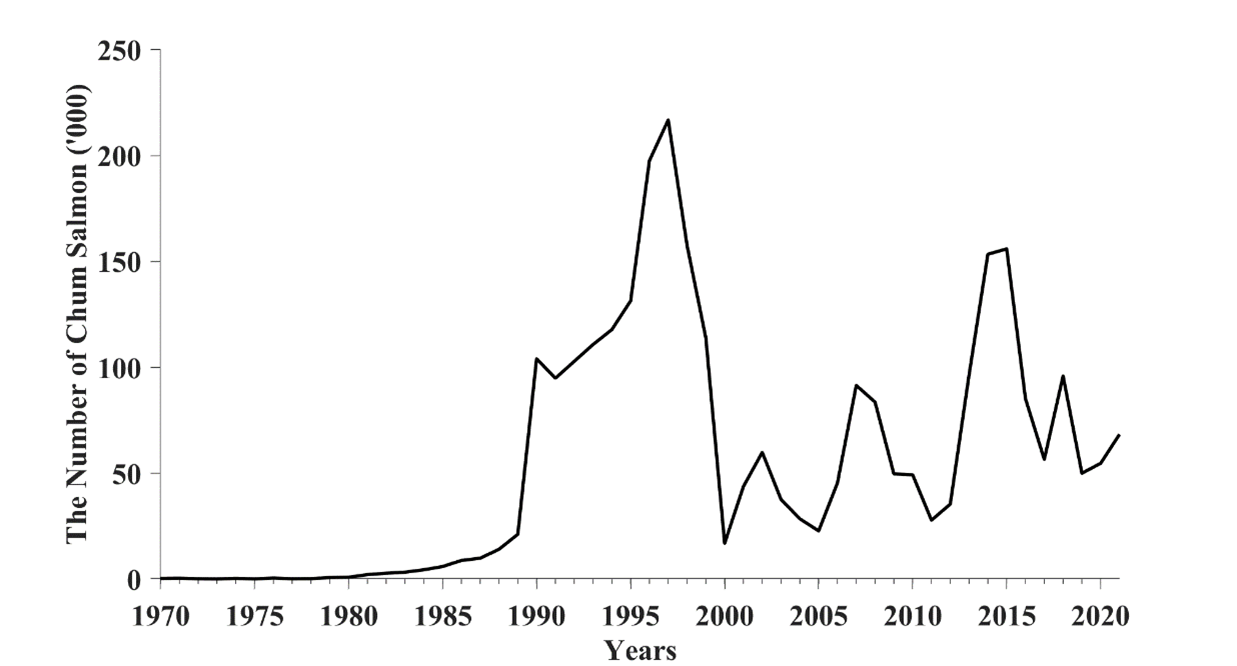

Supplement: S2 Fig — (TIF) [file pone.0317917.s007.TIF]

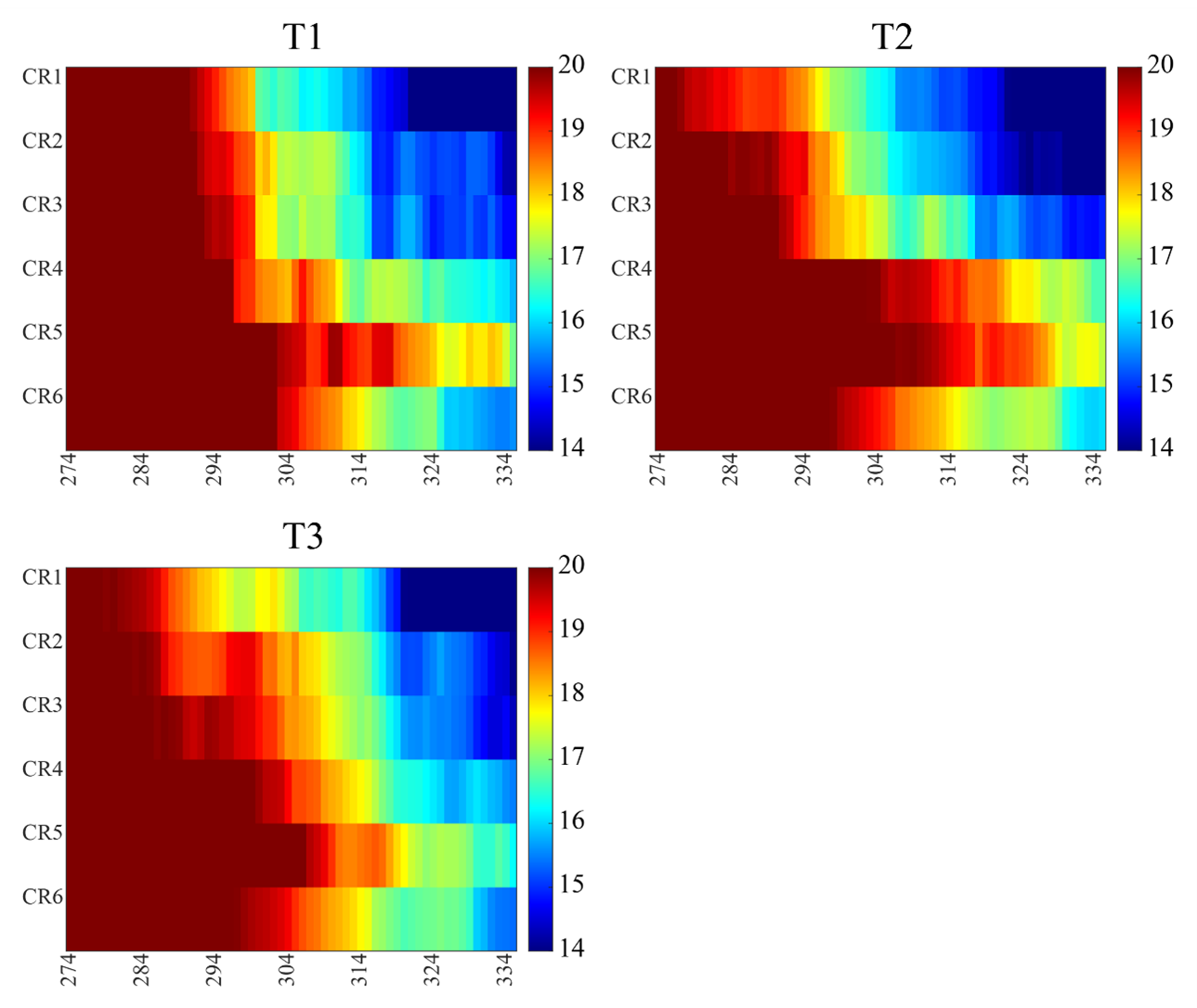

Supplement: S3 Fig — (TIF) [file pone.0317917.s008.TIF]

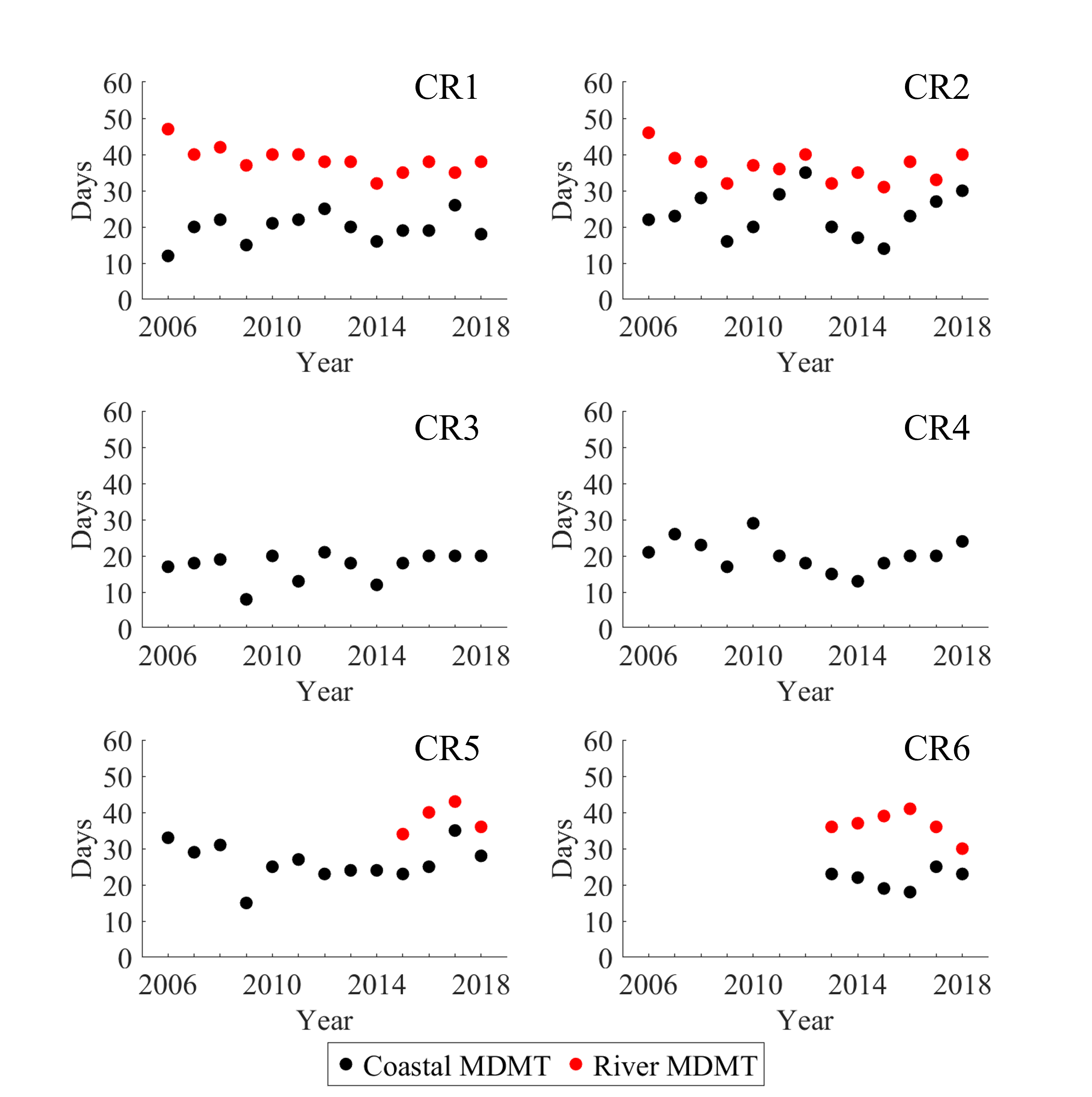

Supplement: S4 Fig — Black dots represent coastal migration timing, and red dots indicate river entry timing. (TIF) [file pone.0317917.s009.TIF]
