# Supplementary material for: Temporal distribution shifts of Chum salmon (Oncorhynchus keta) with sea surface temperature changes at their southern limit in the North Pacific
Source: PLoS One. 2025 Feb 26;20(2):e0317917. doi: 10.1371/journal.pone.0317917 (PMC11864555; doi:10.1371/journal.pone.0317917)
Supplement: Attachment — Submitted filename: Response to Reviewers.docx [file pone.0317917.s011.docx]

**24 Nov 2024**

**Abdul Azeez Pokkathappada, Ph.D.**

**Academic Editor**

***PLOS ONE***

**Dear Editor:**

We wish to re-submit the revised manuscript titled “Temporal distribution shifts of Chum salmon (*Oncorhynchus keta*) with sea surface temperature changes at their southern limit in the North Pacific” The manuscript number is PONE-D-24-40631. This revision and re-submitted manuscript were prepared with Beom-Sik Kim, Hae Kun Jung, Jong Won Park, and Ju Kyoung Kim, with all authors ensuring alignment with the reviewers’ suggestions and the highest quality.

We sincerely appreciate you for taking the time to review our manuscript. Your insightful feedback and suggestions have been incredibly helpful in refining and strengthening our study. The attached response letter includes detailed answers to all reviewer comments and descriptions of the corresponding revisions. Reviewer comments are provided in **black** text, and our responses are highlighted in **blue**.

We hope this revised manuscript meets the high standards of your journal and look forward to your positive consideration.

**Sincerely,**

**Chung Il Lee**

**Department of Marine Ecology and Environment**

**Gangneung-Wonju National University**

**Gangneung 25457, Republic of Korea**

**Tel. +82-33-640-2855**

**E-mail: leeci@gwnu.ac.kr**

**Dear Editor and Reviewers,**

We sincerely appreciate the time and effort you have dedicated to reviewing our manuscript, as well as the insightful feedback and suggestions you provided. Your comments have been invaluable in enhancing the depth and quality of our research.

We carefully reviewed all comments and have made substantial revisions to the manuscript. Specifically, we clarified the research questions and hypotheses, elaborated on the methods and materials, and restructured the figures and tables to enhance clarity. Additionally, we reinforced the ecological and resource management implications of our findings in the Discussion section.

We addressed the reviewers’ suggestions, including adding references, refining sentences, and aligning the study's focus with its scope.

Thank you for your consideration.

**Reviewer #1**

**Overview Comments:**

The authors explore differences between coastal and river migration timing for multiple Chum salmon river systems along the southeastern coast of Korea. They observed differences in coastal migration timing as a function of the difference in SST between northern and southern areas during the period of return migration, as well as differences in river timing as a function of regional temperature conditions. Understanding the potential for behavioural adaption as a result of changing climate is of particular interest for populations such as ESCK Chum salmon, which exist at the southern end of their range.

While the authors pose an interesting research question, the conclusions drawn from their results seemed hypothetical and not always supported by their findings. I also found the level of description with respect to the data sources in the methods to be lacking, which contributed to some confusion in interpreting the results. The display of the results seem inconsistent between the coastal and river timing analyses, with different formatting and labelling applied to similar figures. I also had some concerns with respect to the appropriateness of the statistical analysis and feel like the authors could have investigated the key relationships underlying sea surface temperature and return migration timing using a more comprehensive approach.

Although the research topic is certainly relevant and the results show support for a relationship between ocean temperature conditions and Chum salmon migration behaviour, without major revisions I do not feel that the current article merits publication, in particular due to failure to meet standards related to “Experiments, statistics, and other analyses are performed to a high technical standard and are described in sufficient detail” and “Conclusions are presented in an appropriate fashion and are supported by the data”. More clearly framing their research question may help the authors better refine their choice of statistical analysis and presentation of results to better support their original hypothesis.

For more detail regarding my decision and suggestions for major and minor revisions, please see below.

**Answer:**

We appreciate you for taking the time to review our study and for providing invaluable feedback that has greatly contributed to improving our research. We understand the concerns raised by the reviewer, including the lack of clarity in the research questions, insufficient explanation of data sources, inconsistencies in the presentation of results, and the issues regarding the statistical analysis approach. Based on this feedback, we have made significant revisions to address these issues.

First, to improve the clarity of the research questions, we revised the introduction and objectives sections to clearly present the hypotheses and goals of our study. This has enhanced the logical flow and analytical direction of the manuscript. Additionally, we provided more detailed descriptions of the sea surface temperature (SST) and catch data, including their collection methods and spatial and temporal scope, to strengthen the explanation of the data sources.

In the results section, inconsistencies in the labeling and formatting of visual materials have been addressed. This difference was caused by the different LME model equations that are best for analyzing the difference between coastal arrival times and coastal stay times by SST type. We revised the methods and results sections to clarify these differences and improve reader comprehension.

Regarding the statistical analysis, we re-evaluated the statistical approaches to ensure they appropriately support the main hypotheses of the study. We thoroughly validated the model assumptions and confirmed their suitability. Furthermore, we clarified the limitations of the analytical methods in the discussion section and proposed directions for future research.

Once again, we sincerely appreciate the valuable advice and feedback. We hope these improvements have contributed to significantly advancing the manuscript. Detailed answers to specific questions are provided below.

Q1) I found the description of the underlying data sources in the paper to be lacking. In particular, it was unclear the location, number, and time period over which sea surface temperature data was collected. In addition, it would useful for the authors to address underlying assumptions resulting from the use of catch data as a proxy for abundance. For example, how did they standardise for changes in effort or catchability? I also found it strange that the river locations were referred to generically instead of actually naming, and providing some basics characteristics of, each system. For example, what is the relative return size of the Chum salmon population to each river? Are these all wild stocks? Hatchery? Or a combination? A figure showing historical coastal and river entry timing (e.g. year vs. timing) for each location would have also provided a useful baseline for understanding the underlying differences across locations, and across different sea surface temperature SST “types”.

- A1) Thank you for your valuable feedback. In response to your suggestions, we have added detailed explanations regarding the sea surface temperature (SST) and catch data in the Materials and Methods section (L133–138, L144–146, L159–164). The rivers CR1–CR6 are areas where Chum are annually caught and released as part of hatchery programs. However, we cannot determine the exact proportion of individuals returning to each river that originate from hatcheries versus those that are wild. Additionally, we have included year-by-year data on the MDMT of Chum arriving at coastal waters and entering rivers in S4 Fig. We sincerely appreciate your insightful comments, which have greatly contributed to improving our study. Thank you again.

Q2) While the use of SST “types” was interesting, I was still not clear as to how this particular characterisation of ocean conditions would be preferable to using a numerical indicator such as SST (or SST anomaly) averaged over a relevant time period (e.g. centred around peak historical migration timing). The authors do not clearly characterise how they defined a year as cooling faster or slower than average. I was also concerned about the limited sample size (3-years only) for “Type 1” conditions, and the fact that the timeseries ended in 2018. If the author’s want to continue with the current SST “types” as defined in the current study, I think they need to:

(a) better justify their approach for defining alternative SST “types”

(b) more clearly explain how the metrics to define each “type” was calculated

(c) consider the trade-offs between using a categorical variable as opposed to a numerical representation of ocean temperature conditions

- A2) Thank you for your valuable feedback, which has greatly contributed to improving this study. The ESCK is characterized by the interaction of cold-water masses flowing southward from the north and warm water masses moving northward from the south, resulting in distinct SST distribution types along the coastline. As you mentioned, studying the relationship between SST and Chum migration timing is an interesting research topic, and we have submitted related work to another journal, which is currently under review. In this study, we focused on investigating how Chum returning to Korea respond to these SST distribution types.

The SST types in this study were defined as follows: T1 and T3 represent conditions where the northern and southern areas cool below 20°C around the same time. However, T1 indicates an earlier-than-average cooling, while T3 represents a later-than-average cooling. T2 reflects conditions where the northern area cools faster than the southern area, regardless of whether the cooling is earlier or later than the average. These definitions have been clarified and revised in L164-174.

Q3) I was also unclear on the author’s definition of river entry timing. On L. 180-181, the authors state: “The river entry timing in each region was determined as the period between the MDMT of the ESCK and that of the adjacent river”. I interpreted this to mean that the river timing was being represented as the relative difference between arrival in coastal areas and arrival into the river. I presume this is because river entry timing is a function of both coastal arrival timing AND how long fish delay in coastal areas before entering the river, and the authors were more interested in the latter. i.e. assess whether fish were delaying longer in coastal areas before migrating upstream. However, subsequent wording in the body of the report and in the figures seemed to suggest that the analyses were performed directly on the median arrival timing into the river.

- A3) Thank you for your valuable feedback. Based on your feedback, we have replaced the term "river entry timing" with "coastal residence time" throughout the manuscript (such as L197-198).

Q4) Given that a large part of the focus on the results was on latitudinal differences in responses to changing temperature, particularly for river timing, I also wonder if the authors considered using the latitude of each river mouth as a continuous variable as opposed to the fixed location definitions.

- A4) Thank you for raising an important point. In this study, the "Regions" variable is defined based on latitude, from north to south, effectively reflecting changes in latitude. Additionally, we conducted supplementary analyses comparing the results of using latitude as a continuous variable versus the fixed location definitions (Regions). The results from both approaches were nearly identical, suggesting that the "Regions" variable sufficiently represents latitudinal variation and adequately explains the response patterns based on latitude, which is the main focus of this study. Therefore, we chose to retain the fixed location definitions in the final model to maintain clarity in interpretation.

Q5) The author’s used AIC to select across fixed model types. Another option would have been to apply a step-wise AIC to the most complex model form. Given the small sample sizes in some groupings (e.g. only 3 years of data for SST Type “1”; only 4 locations used for river timing analysis) and the appearance of unequal variances in some of the figures, I wonder if the authors considered any failure of underlying model assumptions for their linear analysis. I also did not see any major advantage in the non-linear analysis in terms of supporting the authors results. The main question the authors appeared to be asking is if the peak river migration varied as a function of SST. This could be done much more simply, and potentially grouped together with the linear analysis by including SST associated with the median timing date as a variable in the model.

- A5) Thank you for your valuable feedback. This study designed to analyze the response of Chum to sea temperature distribution types from a temporal distribution perspective using a simple and clear manner. To achieve this, SST types were treated as categorical variables, focusing on their primary effects rather than exploring additional variables or complex interaction effects. We compared three model equations that included SST types and their interactions with regions, and the best model was selected based on AIC. Employing a stepwise AIC approach to analyze a more complex initial model was considered beyond the scope of this study, as it did not align with our research focus. This approach was chosen to clearly identify the effects of SST distribution types and fulfill the study’s objectives effectively.

Additionally, the interactions between SST types and regions were included as key explanatory variables in the analysis. The exclusion of more complex interactions or additional variables was an intentional decision to maintain consistency in study design and focus. While future research could explore these additional variables and expanded analyses, this study focused specifically on SST distribution types to ensure a clear understanding of their effects.

As you noted, the small sample size in some groups (e.g., three years of data for T1) is acknowledged. To address this, we utilized LME model and verified the normality and suitability during the analysis process, concluding that the model was appropriate for the data.

Q6) I did not find the non-linear analysis to provide a lot of additional useful information. My main interpretation of the resulting figures was that the peak migration tends to be associated with a narrow temperature range for a given river system, with coastal and river timing for northern systems being associated with cooler SSTs than for southern systems. I think this relationship could be illustrated more simply by plotting median timing vs. SST associated with median timing. The histograms are useful in that they illustrate that there is some capacity for adaptation in that the salmon do enter the coastal or river systems over a range of temperatures. But maybe the authors could include a vertical line illustrating the peak timing of migration with a label showing the associated SST. The AIC values for the northern rivers also appeared to be much higher, on average, than for the southern rivers which also raises the question of how useful this analysis was for the northern systems.

- A6) Thank you for your valuable feedback. In this study, the nonlinear regression method was employed to compare the temperature ranges and catch patterns of Chum under different SST distribution types, providing insights into their behavioral strategies in coastal and river environments. Specifically, the analysis aimed to determine whether catches became more concentrated within specific temperature ranges as SST types progressed from T1 to T3, whether higher temperatures were uniquely associated with T2, or whether certain types exhibited evenly distributed catches across a variety of temperature ranges.

The nonlinear analysis revealed that the coastal arrival timing of Chum under T1 and T3 conditions was similar, while T2 showed earlier coastal arrival compared to these conditions. Additionally, under T2 conditions, catches were concentrated in high-temperature environments in both coastal waters and rivers. This suggests the potential for straying behavior as a response to abrupt temperature changes under T2 conditions. Further details on this interpretation are provided in L404-415 of the revised manuscript.

Q7) There was some apparent inconsistency in the presentation of figures throughout the report, with differences in labelling and formatting between figures describing results for coastal vs. river temperatures. While the focus was on trends, and so relative differences are important, the current presentation masks the underlying differences in timing between northern and southern sites, which is referenced by the authors in the text, but would be more clear if the actual timing was displayed.

- A7) Thank you for your valuable suggestions. The differences in labels and formatting in Figures 3, 5, and 6 arose because the optimal LME model equations for explaining the variation in coastal arrival timing and coastal residence time, depending on SST distribution types, were different.

As explained in L199–206 of the manuscript, the optimal model equation for coastal data is MDMT ~ SST_Types + (1|Regions). This indicates that while MDMT differs by region, the extent of its variation across SST distribution types (SST_Types) is consistent across all regions. Consequently, only one figure was presented for coastal data, comparing timing differences across SST types.

In contrast, the optimal model equation for river data is MDMT ~ SST_Types * Regions, which suggests that MDMT varies not only by region but also in response to SST distribution types, with the direction and magnitude of the variation depending on the region. For example, in some regions, MDMT under T1 may be 3 days later than under T2 and 4 days earlier than under T3, whereas in other regions, MDMT under T1 may be 5 days earlier than under T2 and 4 days earlier than under T3. To illustrate this, two types of figures were included: one comparing differences across regions under the same SST type, and another comparing differences across SST types within the same region.

In response to the reviewer’s comments regarding the inclusion of actual timing, we have revised Figures 3, 5, and 6 to display the actual timing data. These revisions aim to improve clarity and better align with the reviewer’s suggestions. Thank you for your insightful feedback.

Q8) I found it challenging to link the patterns of earlier coastal arrival timing in both the north and south under Type T2 conditions to the explanations raised by the authors in the discussion. Some specific examples are provided below.

Q8-1) L. 345 – 348. The authors state that earlier coastal timing under the type T2 SST scenario was because the Chum salmon are trying to avoid migration through abrupt temperature changes. However, it is unclear how southern temperatures would impact the timing into northern coastal areas if the fish migrate from the north into the south. If southern migrants strayed into northern rivers to avoid higher temperatures in the south, then potentially this could impact both abundance and timing in the north but the results are not produced in a clear way to support this argument.

- A8-1) Thank you for your valuable comments. Our study revealed that under T2 conditions, there was a significant temperature difference between the northern and southern regions, which may have had a critical impact on the migration strategies of Chum salmon. Specifically, under T2 conditions, the time required to reach 50% of the coastal catch was shorter compared to other types, and we observed that both coastal and river catches were concentrated in warmer temperature ranges (18–20°C).

Chum salmon returning to spawn in the ESCK region are known to migrate southward, starting from the northern areas (Hong et al., 2022). It is unlikely that Chum in the northern area detects the environmental conditions of the southern area from a distance and halt their southward migration to move toward northern rivers. Similarly, under T2 conditions, it is improbable that Chum migrating southward would reverse their course and return northward after reaching the boundary where cold and warm water masses intersect.

Instead, our study proposes the possibility that some of the Chum migrating southward from the north may move to northern rivers (non-natal rivers) near the boundary of the cold and warm water masses. This behavior could be interpreted as a strategy to avoid stress caused by abrupt temperature changes. We have clearly reflected this possibility in the manuscript in L371-381, to help readers better understand our conclusions and interpretations.

However, this hypothesis is based on statistical analysis results derived from fishing data and has not been directly tested due to the lack of specific data on changes in river-specific abundance or return rates. As suggested in L407–410 of the manuscript, future studies analyzing the relationship between river-specific return rates and SST distribution types will provide a deeper understanding of this phenomenon.

Additionally, to better understand how southern temperatures affect northern timing and abundance, continuous monitoring of abundance and return rate changes between northern and southern areas is necessary. This will be addressed in future studies, which we believe will help overcome the limitations not covered in the current manuscript.

(References)

- <https://doi.org/10.3390/fishes7010027>

Q8-2) L. 374-378. The authors discuss a change in fish abundance in the north vs. the south under type T2 SST conditions, but no analysis was shown to support this result.

- A8-2) Thank you for your feedback. This point has been addressed in response to A8-1.

Q8-3) L. 391-393. Authors state that timing into northern rivers was associated with higher temperatures as overall cooling patterns shifted from earlier to later. However, this does not seem supported by the results in Table S5, where the relationship isn’t significant for CR1 or CR2 under T1, or in CR1 under T2.

- A8-3) Thank you for your meaningful feedback. In this study, the nonlinear regression analysis focuses not only on identifying patterns of coastal arriving and river entry of Chum based on specific temperature ranges but also on understanding how the patterns of Chum migration volume differ by SST distribution types. Specifically, by analyzing whether Chum are concentrated in specific temperature ranges or evenly distributed across various temperature ranges depending on SST distribution types, this study provides key insights suggesting the possibility of Chum moving to non-natal rivers under the T2 environment, where significant temperature differences exist between the northern and southern area.

As you mentioned, we had already noted in the manuscript (see L331-334) that the results for CR1 and CR2 under T1 and T2 conditions in Table S5 were not statistically significant. This suggests that Chum were not predominantly caught in specific temperature zones during these periods and regions. Instead, they may have been distributed more evenly across various temperature ranges or concentrated in multiple zones.

From this perspective, when analyzing the cumulative average catch by temperature range, we observed an increasing trend of Chum being concentrated in higher temperature zones (e.g., 18–20°C) from T1 to T3 across both coastal and river regions. This finding contributes to a better understanding of the relationship between Chum migration patterns and temperature changes, as described in the manuscript.

Q8-4) The authors could have expanded more on how behavioural strategies between northern and southern systems may have been impacted because southern migrants are already dealing with warmer temperatures on average which are closer to the physiological limits for salmon than the conditions experienced by migrants in the north. It would also have been informative to tie the results and discussion back to statements in the introduction relating to improved returns to the ECSK even as environmental conditions have become less favourable. Is this because of adaptability? Or have there also been coincident declines in fisheries harvest contributing to this trend?

- A8-4) Thank you for your question, and I appreciate the opportunity to address it. This study compared the movement and behavioral characteristics of Chum returning to northern and southern areas. Chum returning to northern areas seem adapted to arriving in colder environments with immature gonads, while those returning to southern regions appear to adapt by expending more energy during migration through warmer environments, which accelerates gonad maturation.

As a result, northern populations tend to stay longer in coastal waters before moving to rivers when coastal water are colder. In contrast, southern populations are more likely to take advantage of the cooling water temperatures as a cue to quickly migrate into rivers. Our findings show that the time interval between MDMT at the coastal water and MDMT in the river (referred to as coastal residence time in the re-submitted manuscript) gradually shortened for northern populations from T1 to T3, while it lengthened for southern populations. These patterns indirectly indicate differences in migratory and behavioral strategies between the two regions. This is discussed in detail in L411–422 of the revised manuscript.

4. The article is presented in an intelligible fashion and is written in standard English.

Q9) Paper was difficult to follow in sections due to unclear definitions, changes in flow between methods and results, and inconsistency in figure labelling. For example, the term “region” was sometimes used to describe individual rivers and at other times to describe the northern/southern aggregations of study sites. The term “advanced” timing could also be interpreted as fish arriving either earlier, or later, to a system.

- A9) Regarding the consistency of figure labels, we have addressed this issue as discussed in A7. In terms of terminology, we have revised and reviewed the manuscript to incorporate the reviewer’s suggestions. References to "Northern and Southern regions", "Northern ESCK" and "Southern ESCK" have been adjusted to "Northern area" and "Southern area" for clarity.

Additionally, we acknowledged that the term "advanced timing" could be interpreted in various ways depending on the context. In this study, "advanced timing" specifically refers to the timing that occurs earlier than the average. To ensure clarity, we have replaced this term with "earlier timing compared to" throughout the manuscript, explicitly indicating the reference point for comparison. These changes aim to provide greater precision and prevent potential misinterpretation.

**Reviewer #2**

**Overview comments:**

The article on the temporal distribution shifts of Chum salmon (Oncorhynchus keta) in relation to sea surface temperature (SST) changes provides valuable insights into how marine species respond to climate change. However, several aspects could be strengthened to enhance the study’s rigor and its broader implications.

**Answer :**

Thank you for your valuable feedback and for taking the time to provide insights that have greatly contributed to the improvement of our study. Based on your key comments, we have clarified the focus of the research, addressed areas requiring further explanation, and added discussions on limitations and future research directions.

In particular, we acknowledge the need for a multifaceted approach that includes environmental and ecological factors beyond SST, and we have explicitly identified this as a direction for future research. Additionally, we have provided a detailed rationale for the classification of SST distribution types and the interpretation of results, while incorporating policy implications and regional specificities into the manuscript.

Your feedback has enabled us to make significant improvements to the manuscript. Once again, thank you for your insightful suggestions.

Q1) The focus on analyzing the impact of SST on Chum salmon migration is timely and relevant, yet the study appears to treat SST as a standalone variable without sufficiently considering other influential factors. For instance, changes in ocean currents, salinity, and nutrient availability are critical factors that can also impact the distribution and migration patterns of marine species. By not addressing these potential confounding variables, the study may oversimplify the complex interplay of factors affecting Chum salmon behavior.

- A1) Thank you for providing such valuable insights. As you mentioned, studying the migration timing of Chum by considering SST along with other variables could indeed be an interesting avenue of research. However, this study specifically focuses on the unique environmental characteristics of ESCK, where cold water masses from the north and warm water masses from the south intersect. We aimed to analyze how Chum returning to ESCK responds to SST distribution, with a particular focus on coastal arrival timing and residence time.

Q2) I recommend modifying and updating the first statement of the introduction with given studies as [1,2] “Similar to agricultural ecosystems, climate change also impacts marine ecosystems [3]. Recently, marine ecosystems have been affected…

[1] https://doi.org/10.1016/j.agsy.2024.103994

[2] https://doi.org/10.3389/fenvs.2021.826838

[3] https://doi.org/10.1016/B978-0-12-822373-4.00024-0

- A2) Thank you for your valuable feedback. We have incorporated your suggestion and added the relevant content to L36–37.

Q3) The categorization of SST into three types (T1–T3) provides a structured approach to examining temperature variations. However, the definition of these categories appears somewhat arbitrary, with limited justification for why these specific types were chosen. It would be beneficial to clarify the criteria used to distinguish between rapid and slow cooling, especially given the potential variability in regional oceanic conditions.

- A3) Thank you for your thoughtful comments. In this study, SST types are defined as follows: T1 and T3 indicate cases where both northern and southern areas cool below 20°C around the same time. However, T1 refers to cases where cooling occurs earlier than average year, while T3 refers to cases where cooling occurs later than average year. T2 represents cases where the northern area cools more rapidly than the southern area, regardless of whether the cooling is earlier or later than average. We have revised and clarified these definitions in L164-174.

Q4) While the study identifies a temporal shift in riverine entry timing based on SST changes, the conclusions could be more robust if supported by additional ecological data. For instance, the study could benefit from integrating biological factors such as the age structure of the salmon population, prey availability, and predator pressure, which might influence the observed migration patterns.

- A4) We greatly appreciate your insightful feedback. Interpreting Chum migration patterns from a multifaceted perspective is indeed essential. The timing and patterns of Chum migration are influenced not only by SST but also by various ecological factors, such as the age structure of returning individuals and predator pressure. However, the foundational research and data necessary for quantitatively analyzing or integratively interpreting these factors are currently limited.

In this study, we focused on interpreting migration patterns based on the available data and prior research, emphasizing Chum responses to SST distribution types. SST is a major environmental factor that directly affects the physiological state of Chum, particularly their maturation rate and energy expenditure, as demonstrated by numerous previous studies. Specifically, warmer waters are known to increase energy expenditure and induce physiological stress, potentially altering migration timing and routes.

Future studies require an integrated approach, including ecological factors, to better understand Chum migration and behavior patterns. For example, data on age structure, energy state, maturity, and food web dynamics will be collected and compared with water temperature for more detailed analyses. These additional studies are expected to provide deeper insights into the complex mechanisms of Chum migration.

Q5) The lack of significant differences in coastal arrival timing between the T1 and T3 scenarios raises questions about the sensitivity of the analysis. The study could delve deeper into why these changes in SST, despite being categorized as rapid or slow cooling, did not produce observable effects on salmon arrival. A discussion on the potential buffering mechanisms that Chum salmon might possess against temperature fluctuations would provide a more comprehensive understanding.

- A5) Thank you for your valuable feedback. Firstly, as cold water is consistently distributed in the bottom layer throughout the year from the northern to the southern parts of the ECK, it is assumed that there is no significant difference in the migration process of Chum moving southward when the surface environmental conditions, such as T1 and T, do not vary greatly between the northern and southern areas (this explanation has been added to the main text at L358-360).

However, in scenarios like T2, where the temperature difference between the northern and southern areas is significant, gonadal dysfunction may occur. As explained in L382-406, Chum may adopt migration strategies to avoid abrupt temperature changes during their journey from the north to the south.

Q6) The article presents its results as if they are directly applicable to predicting future climate change impacts on Chum salmon distribution. However, it does not account for the fact that SST is only one of many factors affected by global warming. The study's findings would be more meaningful if contextualized within broader climate change projections, such as ocean acidification or changes in seasonal precipitation patterns, which can also alter habitat suitability for salmon .

- A6) Thank you for your valuable feedback. As you mentioned, SST is a significant factor influencing the migration and movement timing of Chum, but we recognize that it is just one of many factors associated with climate change. For instance, seasonal rainfall changes determine the influx of freshwater into coastal areas, potentially affecting Chum’s ability to navigate from coastal to riverine environments, thereby influencing their migration and distribution characteristics. Thus, predicting changes in Chum distribution based on this study’s findings requires integrated research that considers other climate change factors in addition to SST variations.

However, this study specifically aimed to investigate how the changes in SST distribution in ESCK, a region characterized by the intersection of cold-water masses from the north and warm-water masses from the south, influence the migration of Chum returning to Korea. To achieve this, the interaction effects of SST distribution types and regions were selected as the main explanatory variables. Additional continuous variables or complex interactions were not included to maintain consistency in the research design and focus. While future studies could explore analyses incorporating these additional variables, this study centered on SST distribution types to clearly understand their specific effects.

Q7) Additionally, while the study claims that the findings are useful for predicting changes in Chum distribution and resources in the North Pacific, it does not provide specific recommendations for fisheries management or conservation strategies. This is a missed opportunity to connect the research to actionable policy measures, especially given the importance of Chum salmon for both ecological systems and coastal communities.

- A7) Thank you for your valuable comments. As you pointed out, the findings of this study provide important insights for the development of Chum resource management and conservation strategies. Currently, uniform criteria for Chum fishing bans are applied across all regions. However, our results suggest that these fishing ban periods could be adjusted based on environmental conditions and regional characteristics. This point has been specifically addressed in L448–459, where we emphasize the necessity of region-specific management strategies.

Additionally, as global warming, Chum resource management strategies are shifting from increasing release numbers to improving early life stage survival rates. In the past, releases were conducted on fixed schedules without considering coastal environmental variability or the specific environmental characteristics of each region. Moving forward, release timing should be adjusted based on regional environmental conditions and sea surface temperatures. Expanding research to include early life stages could offer crucial insights for sustainable resource management and ecosystem health. These points have also been specifically detailed in L460–465.

Q8) Lastly, the article could be improved by expanding its geographical scope. Focusing solely on the eastern and southern coastal waters of Korea limits the generalizability of the results. Including data from other parts of the North Pacific could provide a more holistic view of the species’ adaptation strategies across their entire southern distribution range. some regional studies are suggested to incorporate and validate with your results in discussion section.

https://doi.org/10.3390/atmos14010079

https://doi.org/10.1088/1748-9326/ac8fa6

https://doi.org/10.3389/fenvs.2023.1228817

https://doi.org/10.3390/w15132420

- A8) Thank you for your insightful comments. This study aims to understand how Chum respond to changes in the distribution of warm and cold water masses, focusing on the unique environmental characteristics of ESCK, where the boundaries between the warm and cold water masses are distributed. We analyzed how the coastal arrival timing and coastal residence time differ according to SST distribution type.

Chum are reportedly found in the North Pacific, spanning Korea, Japan, Russia, the United States, and Canada. However, the unique interplay between these water masses is observed in limited areas, such as the East Sea of Korea and the Pacific side of Honshu and Hokkaido in Japan. There is limited understanding of how Chum responds to their migration timing and strategies in response to SST distribution patterns in these specific areas. Nevertheless, the relationship between coastal surface temperatures and the migration or behavioral patterns of Chum in such areas has been addressed in numerous studies, as discussed in L105–110 and L423–426 of this manuscript. Additionally, comparisons between the surface temperatures and return patterns of Chum in ESCK and other areas are being examined in a separate study currently under review in another journal.
